# Has doxycycline, in combination with anti-malarial drugs, a role to play in intermittent preventive treatment of *Plasmodium falciparum* malaria infection in pregnant women in Africa?

**DOI:** 10.1186/s12936-018-2621-x

**Published:** 2018-12-14

**Authors:** Tiphaine Gaillard, Manon Boxberger, Marylin Madamet, Bruno Pradines

**Affiliations:** 1 Fédération des Laboratoires, Hôpital d’Instruction des Armées Desgenettes, Lyon, France; 2 Unité Parasitologie et Entomologie, Département Microbiologie et Maladies Infectieuses, Institut de Recherche Biomédicale des Armées, 19-21 Boulevard Jean Moulin, 13005 Marseille, France; 3 Aix Marseille Univ, IRD, SSA, AP-HM, VITROME, IHU Méditerranée Infection, Marseille, France; 4 Centre National de Référence du Paludisme, Institut de Recherche Biomédicale des Armées, Marseille, France

**Keywords:** Antibiotics, Doxycycline, Malaria, *Plasmodium falciparum*, Anti-malarial drug, Resistance, Intermittent Preventive Treatment, Pregnancy

## Abstract

According to the World Health Organization (WHO), *Plasmodium falciparum* malaria during pregnancy is responsible for deleterious consequences for the mother and her child. The administration of intermittent preventive treatment (IPTp) with sulfadoxine–pyrimethamine (SP) at each antenatal care visit as early as 13 weeks of gestation until the time of delivery is a strategy that is currently recommended by WHO for the prevention of malaria in pregnancy. However, the emergence and the spread of the resistance to SP in Africa raise the question of the short-term effectiveness of the strategy. Dihydroartemisinin–piperaquine 120 mg/960 mg once a day for 3 consecutive days administered at least three times during the pregnancy might be an option for IPTp. The combination of 200 mg of doxycycline once a day for 3 consecutive days seems to be a good option to retard the emergence and the spread of resistance to artemisinin-based combination therapy (ACT) in Africa and improve the effectiveness of ACT in term of preterm births, neonatal morbidity and mortality. Contrary to preconceived ideas, scientific and medical data suggest that the risk of congenital malformations in the fetus or of tooth staining in infants whose mothers take doxycycline and hepatotoxicity during pregnancy is very low or non-existent. Additionally, the use of doxycycline during the first and second trimesters leads to an increase in gestational age at delivery, a decrease in the number of preterm births and a reduction in neonatal morbidity and mortality due to the beneficial antimicrobial activity of doxycycline against other infections during pregnancy. Furthermore, doxycycline has anti-malarial properties and is already recommended as prophylaxis for travellers and for treatment of falciparum malaria in combination with other anti-malarial drugs.

## Background

According to the World Health Organization (WHO), *Plasmodium falciparum* malaria during pregnancy is responsible for deleterious consequences for the mother and her child, among which fetal growth restriction, prematurity and stillbirth contribute to perinatal and neonatal mortality [1]. In areas where the intensity of transmission is moderate to high, leading to higher levels of acquired immunity, most *P. falciparum* malaria infections during pregnancy remain asymptomatic and frequently undiagnosed and untreated [2, 3]. In these areas, the administration of intermittent preventive treatment (IPTp) with sulfadoxine–pyrimethamine (SP) is a strategy that is currently recommended by the WHO for the prevention of malaria in pregnancy. IPTp with SP is recommended at each antenatal care visit as early as 13 weeks of gestation until the time of delivery [4]. The WHO has recommended the administration of at least three curative doses of SP during pregnancy, and irrespective of malaria infection status. However, only 19% of pregnant women received at least three doses of SP among the 23 African countries reporting IPTp to WHO in 2016 (6% in 2010, 13% in 2014, 18% in 2015) [5]. In sub-Saharan Africa, IPTp with SP has been shown to reduce maternal anaemia [6], low birth weight [7–10] and perinatal mortality [8]. Additionally, IPT with SP protects not only against malaria and adverse birth outcomes, but also against sexually transmitted and reproductive tract infections that could be responsible for preterm delivery [11]. However, the emergence and the spread of the resistance to SP in Africa raise the question of the short-term effectiveness of the strategy [12].

In Burkina Faso, the use of SP in IPTp was associated with an increased prevalence of mutations involved in pyrimethamine resistance [13]. The efficacy of IPTp with SP to treat and prevent malaria infection has been compromised in areas with > 90% prevalence of the mutations involved in sulfadoxine resistance [10]. Few alternatives are currently available, among which dihydroartemisinin–piperaquine may be the most promising candidate for IPTp [14, 15]. However, resistance to dihydroartemisinin–piperaquine has emerged in Cambodia [16–18] and may rapidly spread to Africa, compromising its use for IPTp. Another alternative is to use triple combination, and more particularly triple artemisinin-based combination therapy (ACT), to retard the emergence and the spread of resistance to ACT in Africa [19]. Doxycycline might be this third partner drug for IPTp to prevent and treat malaria in pregnant women.

## Doxycycline is an antibiotic with anti-malarial properties

Doxycycline is already recommended at a dose of 100 mg/day as malaria prophylaxis for travellers in Africa [20–23]. It also recommended for the treatment of falciparum malaria in combination with quinine in the event of ACT unavailability or clinical failure with artesunate or with artesunate in the treatment of severe malaria [24–26]. The slow schizonticidal action of doxycycline justifies its association with a fast schizonticide [27, 28]. Additionally, recent work showed that doxycycline prevented cerebral malaria in the experimental model using *Plasmodium berghei* by attenuation of brain inflammation and reduction of expression of tumour necrosis factor and chemokines [29].

## Doxycycline is efficacious against pathogens that are responsible for urogenital infections, which are deleterious for the fetus

Oral doxycycline treatment (200 mg the first day followed by 100 mg for 6 days) during the first and second trimesters increased the duration of gestation and decreased the number of preterm births [30]. This result can be explained by the beneficial antimicrobial activity of doxycycline against infections during pregnancy. Doxycycline is effective against *Ureaplasma* spp., *Mycoplasma* spp. [31, 32] and *Chlamydia trachomatis* infection [33], that are responsible of genitourinary infections, which are responsible of spontaneous preterm labour, prematurity with infant with low birth weight, stillbirth or neonatal death [34]. Additionally, preterm birth, neonatal sepsis and bacterial meningitis are also associated with maternal rectovaginal colonization by group B *Streptococcus* [35] and *Escherichia coli* [36, 37]. Although resistance of these pathogens to doxycycline or tetracycline has been observed in Africa (16.9% of 1041 *Streptococcus pneumoniae* isolates in Kenya, 54.5% in comparison to 86.7% for ampicillin for *Escherichia coli*) [38, 39], doxycycline remains efficacious.

## Doxycycline is contraindicated during pregnancy

Doxycycline is contraindicated during pregnancy by comparison with other tetracyclines, such as oxytetracycline, that show teratogenic risk to the fetus during the second trimester [20, 40]. However, the FDA (Food and Drug Administration) approved doxycycline for the treatment of pregnant women after exposure to biothreat agents, including *Yersinia pestis*, *Bacillus anthracis* and *Francisella tularensis* [41].

## Potential teratogenicity of doxycycline

The first argument to contraindicate doxycycline during pregnancy is based on its potential adverse teratogenic effects. The teratogenicity after doxycycline exposition in pregnant woman is not based on any evidence [40]. The use of oral oxytetracycline in pregnant women in the second month of gestation presented an increased risk of having infants with congenital abnormalities [odds ratio (OR) of 12.9, 95% confident interval (CI) 3.5–83.5] [42]. However, very few studies have addressed the potential adverse teratogenic effects of doxycycline. Of 18,515 pregnant women who had an infant with a major congenital anomaly (including neural tube defect, cleft lip or palate, cardiovascular malformations, hypospadias, clubfoot), 56 (0.3%) were treated with doxycycline during 6 to 14 days during pregnancy, while 63 mothers exposed to doxycycline out of 32,804 pregnant women (0.19%) had healthy infant [43]. Although a significant difference (p = 0.01), the authors concluded that doxycycline shows very little, if any, risk of congenital malformations in infants because there was no significant difference of exposure to doxycycline during the second and third months of pregnancy, the period of embryogenesis the most sensitive for major congenital anomaly. A limitation of the study is the small size of pregnant women exposed to doxycycline (25 mothers during the second and third months, 119 during the whole pregnancy). On 1843 infants whose mothers had received a 10-day treatment with doxycycline (220 mg/day) during pregnancy for biothreat agents exposure-related scenarios, the proportion of infants with malformations was lower in the group of infants with fetal exposure to doxycycline (2.5%) compared to unexposed infants (2.9%) [44]. Most of the mothers received doxycycline during the first trimester of gestation, the most critical period for teratogenicity, suggesting that there was no increased risk to the foetus following doxycycline use during pregnancy. No congenital anomaly was detected in a small sample size of 43 children at 1 year of age after treatment of their mothers with 10-days doxycycline for *Mycoplasma* infections during the first trimester of gestation [31]. Molgaard-Nielsen and Hviid showed that the use of doxycycline or tetracycline during the second month of pregnancy led to increased risk of cleft lip or cleft palate [45]. However, this conclusion is based on only two cases of tetracycline exposure. Morever, the pregnant women were also exposed to other drugs in the second gestational month. In a national study on birth anomalies, there was no significant association between exposure to tetracyclines during the first months of pregnancy (46 mothers) and the presence of orofacial clefts [adjusted odds ratio (AOR) of 2.0, 95% CI 0.6–6.7] [46]. Additionally, the recommendation for the use of doxycycline in combination with steroids in in vitro fecundation as per-implantation prophylaxis to protect the embryo is favoured by its very weak toxicity against embryo [47, 48]. The overall evidence suggests that the risk of congenital malformations in infants whose mother received doxycycline is very low [31, 41, 43–46, 49–51].

## Potential yellow tooth discolouration

Concerning the adverse dental effects, tooth discolouration is the main adverse effect associated with tetracycline in pregnancy, by formation of a complex tetracycline-calcium orthophosphate that is irreversibly incorporated into teeth during tooth calcification that occurs at the beginning of the second trimester of gestation [52–55]. The prevalence of tooth discolouration due to tetracycline exposure during pregnancy was estimated at 3–6% [56]. However, no study has evaluated the risk of permanent yellow tooth discolouration in infants after doxycycline use during pregnancy. The only studies on the potential effect on tooth of doxycycline were assessed on children under 8 years of age treated with doxycycline and reported the safety of doxycycline [40, 57]. Three studies found no significant differences in the incidence of tooth staining between doxycycline-treated cases and non-doxycycline exposed children [58–60]. A recent study on 36 children ranged between 0.6 to 7.9 years-old treated at 10 mg/kg/day during 2 to 28 days also reported that doxycycline does not lead permanent tooth discolouration [61].

## Potential hepatotoxicity of doxycycline

Finally, doxycycline is afflicted with adverse hepatic effects for which no evidence can be found, in contrast to the well-documented hepatotoxicity of tetracycline [62–64]. Some cases of fatal hepatotoxicity after doxycycline administration were reported only in combination with other hepatotoxic agents. Hepatic failure was observed in a patient 5 days after this patient received doxycycline, paracetamol and acetylsalicylic acid, two hepatotoxic drugs [65]. A case of lethal hepatitis was reported in a patient with acute *Mycoplasma pneumoniae* infection and was likely caused by the combination of levofloxacin, doxycycline and naproxen [66]. Among 148,879 patients, 32 serious adverse events (renal toxicity and ventricular arrhythmias) were reported in patients exposed to doxycycline and no hepatic toxicity was observed in 136,554 patients [67]. In a study analysing 3377 patients exposed to either tetracycline or doxycycline and presented a case of hepatotoxicity (89% of toxic hepatitis, 6% of acute necrosis and 5% of hepatic coma), tetracycline was found to be associated with an increased risk of liver damage, with an OR of 3.70 (95% CI 1.19–11.45) [68]. Conversely, the authors concluded that doxycycline did not present a risk of hepatic toxicity, with an OR of 1.49 (95% CI 0.61–3.62]. In a retrospective comparative study on treatment of uncomplicated falciparum malaria with quinine–doxycycline and artemether–lumefantrine, quinine–doxycycline group showed a lower proportion of liver enzymes abnormalities (elevation of transaminases, alkaline phosphatase and total bilirubin) (5% versus 42%, p < 0.01) [69]. There is no evidence that doxycycline is associated with adverse hepatic effects.

## Conclusion

Scientific and medical data suggest that the risk of congenital malformations in the fetus or of tooth staining in infants whose mothers take doxycycline and hepatotoxicity during pregnancy is very low or non-existent. Additionally, the use of doxycycline during the first and second trimesters leads to an increase in gestational age at delivery, a decrease in the number of preterm births and a reduction in neonatal morbidity and mortality due to the beneficial antimicrobial activity of doxycycline against other infections during pregnancy.

Furthermore, doxycycline has anti-malarial properties and is already recommended as prophylaxis for travellers and for treatment of falciparum malaria in combination with other anti-malarial drugs. Doxycycline might be an excellent candidate associated with SP in IPTp. Doxycycline at a dose of 200 mg could be administered with 1500 mg/75 mg of SP as soon as the 13 weeks of gestation, that limits the occurrence of potential teratogenic effects (the critical period is during the second and third months). Resistance to SP raises the question of the short-term effectiveness of IPTp strategy with SP. Dihydroartemisinin–piperaquine 120 mg/960 mg once a day for 3 consecutive days administered at least three times during the pregnancy might be an option for IPTp. The combination of 200 mg of doxycycline once a day for 3 consecutive days seems to be a good option to retard the emergence and the spread of resistance to ACT in Africa and improve the effectiveness of ACT in term of preterm births, neonatal morbidity and mortality.

## Abbreviations

ACT: artemisinin-based combination therapy
AOR: adjusted odds ratio
50% CI: 50% confident interval
FDA: Food and Drug Administration
IPTp: intermittent preventive treatment in pregnancy
OR: odds ratio
SP: sulfadoxine–pyrimethamine
WHO: World Health Organization
